# Discovering the key genes and important DNA methylation regions in breast cancer

**DOI:** 10.1186/s41065-022-00220-5

**Published:** 2022-01-21

**Authors:** Yan-Ni Cao, Qian-Zhong Li, Yu-Xian Liu, Wen Jin, Rui Hou

**Affiliations:** 1 Laboratory of Theoretical Biophysics, School of Physical Science and Technology, Inner Mongolia University, No.235 West Daxue Street, Saihan District, Hohhot, 010021 P.R. China; 2 The State Key Laboratory of Reproductive Regulation and Breeding of Grassland Livestock, Inner Mongolia University, Hohhot, 010070 China

**Keywords:** Breast cancer, DNA methylation, Oncogene, Tumor suppressor gene, Biomarker

## Abstract

**Background:**

Breast cancer is the malignant tumor with the highest incidence in women. DNA methylation has an important effect on breast cancer, but the effect of abnormal DNA methylation on gene expression in breast cancer is still unclear. Therefore, it is very important to find therapeutic targets related to DNA methylation.

**Results:**

In this work, we calculated the DNA methylation distribution and gene expression level in cancer and para-cancerous tissues for breast cancer samples. We found that DNA methylation in key regions is closely related to gene expression by analyzing the relationship between the distribution characteristics of DNA methylation in different regions and the change of gene expression level. Finally, the 18 key genes (17 tumor suppressor genes and 1 oncogene) related to prognosis were confirmed by the survival analysis of clinical data. Some important DNA methylation regions in these genes that result in breast cancer were found.

**Conclusions:**

We believe that 17 TSGs and 1 oncogene may be breast cancer biomarkers regulated by DNA methylation in key regions. These results will help to explore DNA methylation biomarkers as potential therapeutic targets for breast cancer.

**Supplementary Information:**

The online version contains supplementary material available at 10.1186/s41065-022-00220-5.

## Background

Breast cancer is the most commonly diagnosed cancer. Worldwide, breast cancer is the leading type of cancer in women, there were about 2.26 million newly diagnosed female breast cancer cases and about 0.68 million death in 2020 [1]. The mortality rate of breast cancer has been decreased compared with the past, which is attributed to the early diagnosis of breast cancer and the improvement of the level for surgery, radiotherapy, and chemotherapy [2–5]. In addition, new targeted drug therapies have significantly improved the survival of breast cancer patients. However, the target drugs for breast cancer are relatively lacking [6, 7]. Therefore, it is important to find new target genes related to the pathogenesis of breast cancer.

Epigenetics is a heritable variation that can cause changes in gene expression [8]. DNA methylation is considered a biomarker for epigenome analysis [9, 10]. Many studies have reported that DNA methylation can affect gene expression, which is an important factor in the development and progression of cancer [11]. Whole-genome hypomethylation and gene-specific hypermethylation were associated with malignant tumors [9, 12]. In particular, hypermethylation of tumor suppressor genes (TSGs) can lead to cancer development [13–15]. For example, *CAV1* [16], *CDH13* [17], *ID4* [18], and *SCGB3A1* [19] are epigenetically regulated by DNA hypermethylation in breast carcinogenesis. The hypermethylation in promoters of *APC*, *SFRP1*, *SFRP2*, *SFRP5*, *WIF1*, *DKK3*, *ITIH5*, and *RASSF1A* [17] are associated with the development of breast cancer, and studies have found that *APC* and *RASSF1A* are common epigenetic biomarkers for early detection of breast cancer [20–22]. Experiments have demonstrated that abnormal DNA methylation in the promoter can down-regulate the gene expression of the *YAP* gene for breast cancer patients [23]. In our previous work, we also found that some key hypomethylation sites in enhancer regions and key hypermethylation sites in CpG islands are used to regulate the expression of key genes, such as oncogenes *ESR1* and *ERBB2*, and TSGs *FBLN2*, *CEBPA*, and *FAT4* [24].

However, despite the significant progress were made in the methylation changes of breast cancer, many questions remain unanswered. Here, we explored the relationship between abnormal DNA methylation in different regions and the differential expressions of genes and found the key regions where DNA methylation abnormalities lead to changes in gene expression. Finally, we discovered 18 key genes related to breast cancer and confirmed that the genes are related to the prognosis of breast cancer.

## Results

### Abnormal DNA methylation distribution in up-regulated hypomethylation and down-regulated hypermethylation genes

**Fig. 1:**
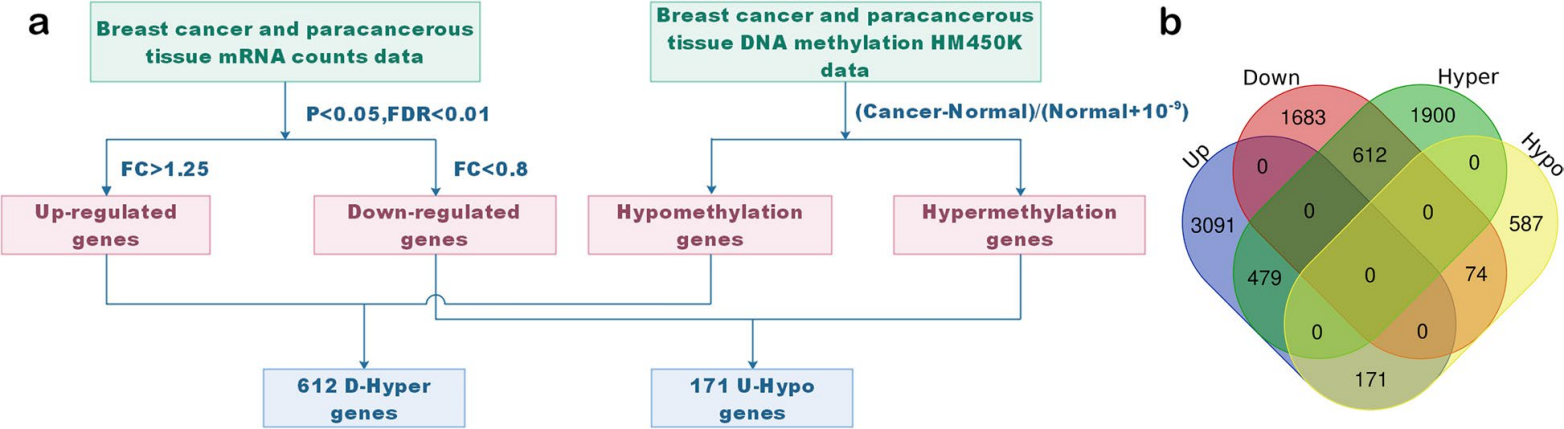
The analysis of aberrantly methylated in the promoter region and differentially expressed genes. **a** The flowchart of D-Hyper and U-Hypo genes. **b** Overlap analysis of up-regulated, down-regulated expression genes and hyper-methylation, hypomethylation genes, up represents up-regulated genes in cancer tissues, down represents genes down-regulated in cancer tissues, hyper represents hypermethylated genes in cancer tissues, and hypo represents hypomethylated genes in breast cancer tissues

The study design flowchart is shown in Fig. 1a. By analyzing gene expression data, we found that 3741 genes were significantly up-regulated, and 2369 genes were significantly down-regulated in breast cancer tissues. By computing the DNA methylation data in the promoter regions of genes, it was found that 2991 genes were hypermethylated and 832 genes were hypomethylated in breast cancer tissues. Because the level of DNA methylation in the promoter region has a negative regulatory effect on gene expression [25], therefore, through the intersection of genes, we found that 171 genes were up-regulated and hypomethylated (U-Hypo), 612 genes were down-regulated and hypermethylated (D-Hyper) in breast cancer tissues (Fig. 1b).

**Fig. 2:**
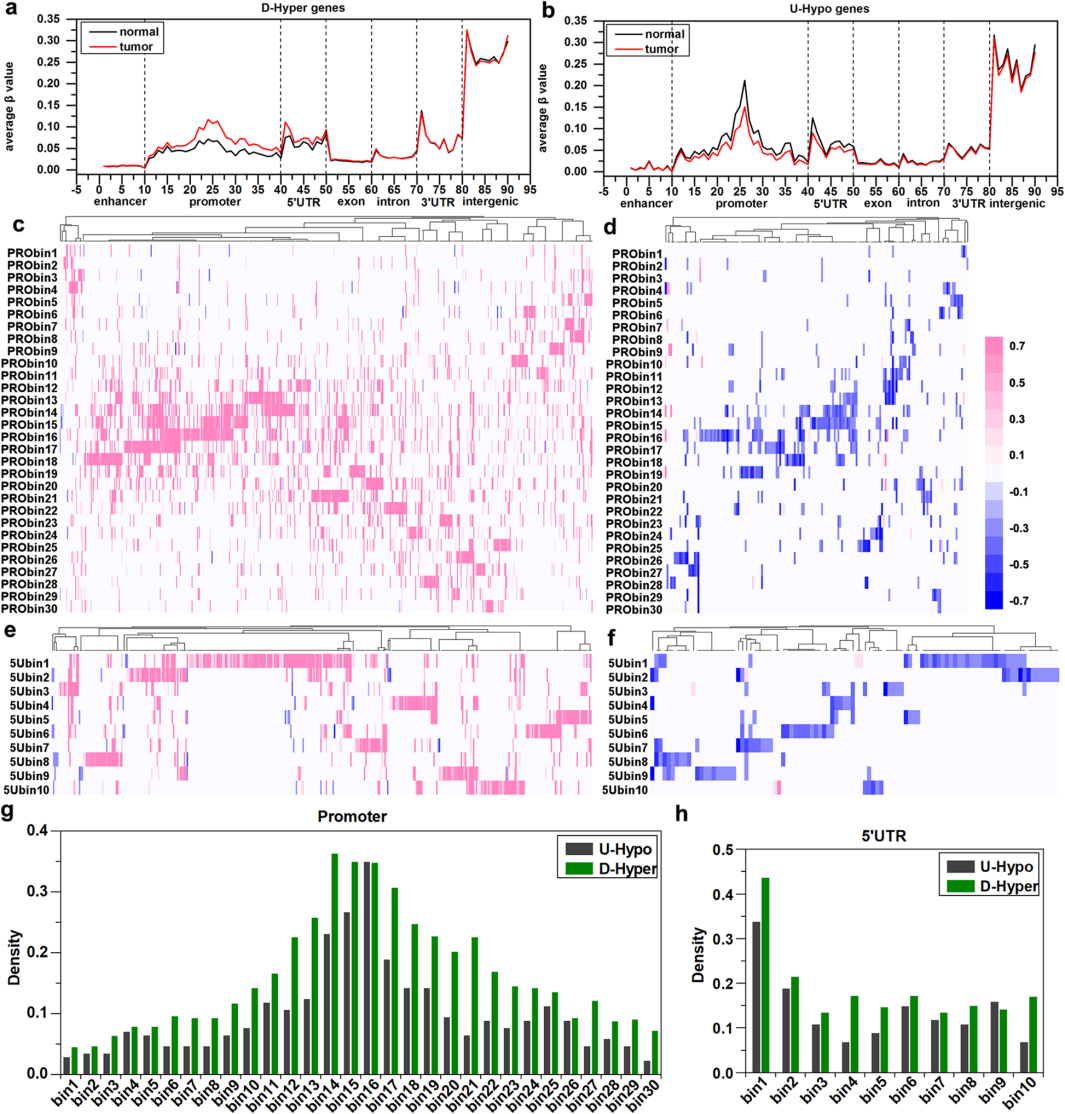
DNA methylation profiles of 612 D-Hyper, 171 U-Hypo genes. **a**, **b** The distribution of DNA methylation in the functional regions of D-Hyper, U-Hypo genes, respectively. Wherein the ordinate is the average *β* value of the CpG site in each bin, and the abscissa is the bin of each functional region. **c**, **d** The distribution of *RD* value in the promoter (PRO) of the D-Hyper and the U-Hypo genes. **e**, **f** The distribution of *RD* value in the 5’UTR (5U) region of the D-Hyper and the U-Hypo genes. Pink means that the DNA methylation value in breast cancer tissues is higher than that in paracancerous tissues, and blue opposite. **g**, **h** The distribution density of aberrant methylation genes in the promoter and 5’UTR regions

To understand the DNA methylation characteristics of these genes and whether DNA methylation has also changed in other regions, we analyzed DNA methylation distribution in the enhancer, promoter, 5’UTR, exon, intron, 3’UTR, and intergenic regions of U-Hypo and D-Hyper genes between cancer and paracancerous tissues (Fig. 2). It can be seen from Fig. 2a, there were no significant changes of the DNA methylation levels in the exons, introns, 3’UTR, and intergenic regions between cancer tissues and paracancerous tissues. The methylation levels in the promoter regions and 5’UTR regions of the D-Hyper genes were significantly higher in cancer tissue than that in paracancerous tissues. Notably, there were significant differences in the 22nd bin (− 400 bp, − 300 bp), 24th bin (− 200 bp, − 100 bp) and 26th bin (0 bp, 100 bp) regions near TSS, and the most difference of methylation occurs in the 24th bin (− 200 bp, − 100 bp). As it can be seen from Fig. 2b, the U-Hypo genes also showed a significant difference of methylation occurs in the promoter and the 5’UTR regions, but the methylation level of the cancer tissues was lower than the methylation level of the paracancerous tissues. The most obvious difference occurs in the 26th bin (0 bp, 100 bp) of the promoter region.

To further investigate the effect of DNA methylation in the promoter and 5’UTR region on gene expression, we calculated the relative difference of DNA methylation between cancer tissues and paracancerous tissues for each bin in each D-Hyper or U-Hypo gene, and results were shown by heat maps (Fig. 2c, d, e, and f). Abnormal DNA methylations were mainly enriched in 14 ~ 17th bins (− 200 bp, 200 bp) of the promoter region for the D-Hyper genes and the U-Hypo genes (Fig. 2c, d, and g), indicating the DNA methylation in 200 bp upstream and downstream of TSS have the obvious difference between cancer tissues and paracancerous tissues. In the 5’UTR region (Fig. 2e, f, and h), the main changes of DNA methylation were significantly enriched in the 1st bin for the D-Hyper genes and the U-Hypo genes.

### The characteristics of abnormal methylation in differentially methylated enhancers (DME) genes

To study the effect of the DNA methylation level in specific regions of enhancers on the gene expression in breast cancer. By using a comprehensive model (Fig. 8), we selected genes (162 DME-gene pairs) with abnormal DNA methylation in the enhancer region, while no abnormal DNA methylation in the promoter region, and the abnormal DNA methylation in the enhancer region is negatively correlated with gene expression (Supplementary Table). Since some genes are regulated by multiple enhancers, 162 DME-gene pairs are corresponding to 154 genes.

**Fig. 3:**
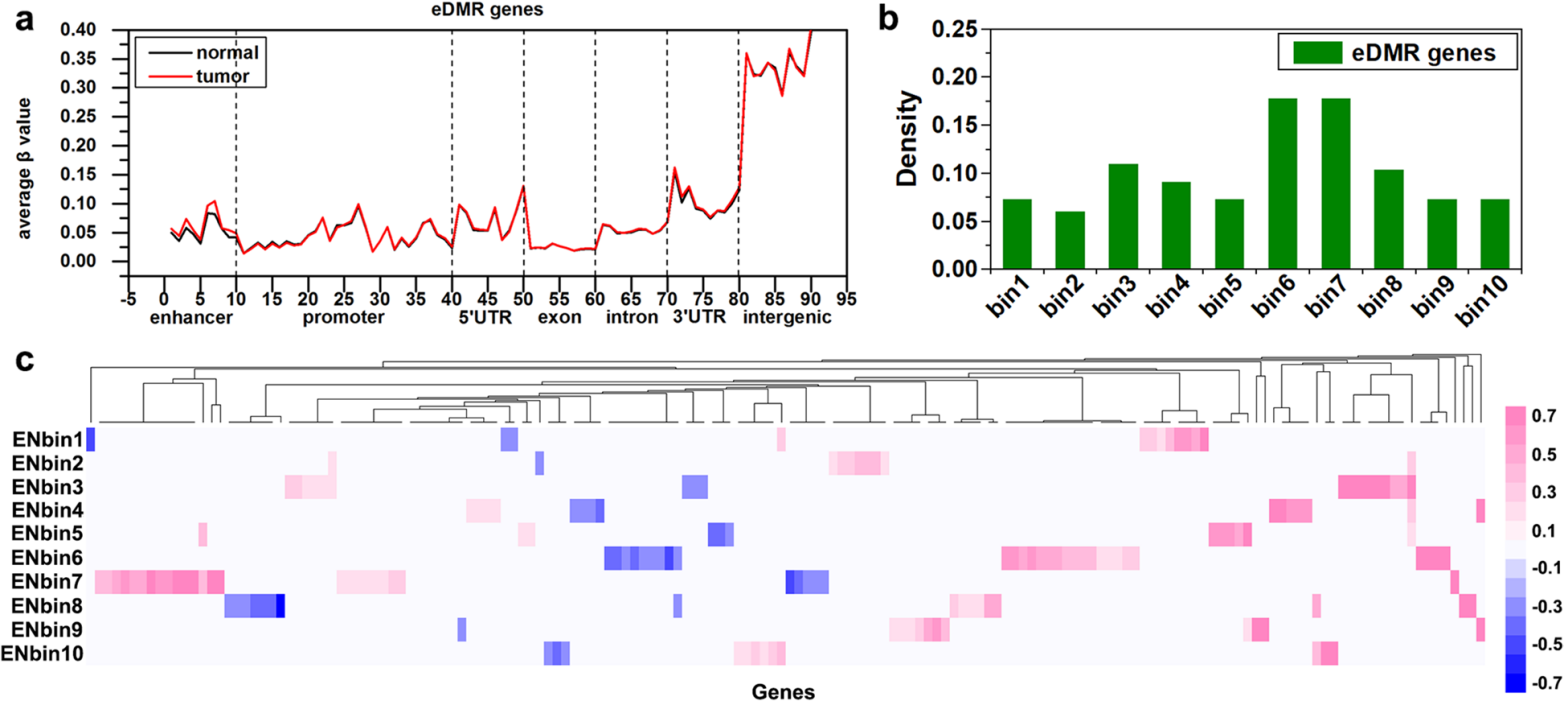
DNA methylation profiles of DME genes. **a** The distribution of DNA methylation in the functional regions of the DME gene set, wherein the ordinate is the average *β* value of the CpG site in each bin, and the abscissa is the bin of each functional region. **b** The distribution density of aberrant methylation in the enhancer. **c** The distribution of *RD* value in the enhancer (EN) of DME genes

To understand the DNA methylation characteristics in the enhancer and other regions of the DME genes, we analyzed the DNA methylation profile of the 154 DME genes. We could conclude that the DNA methylation was different in the enhancer region of DME genes, but the level of DNA methylation had no significant difference in other regions between breast cancer and paracancerous tissues (Fig. 3a). From the differential DNA methylation gene density distribution of the enhancer region, it can be seen that the differential methylation of DME genes was mainly enriched in the 6th and 7th bins (Fig. 3b). Further, we analyzed the differential methylation levels of each bin of the enhancer region in DME genes. There were more hypermethylated genes than the hypomethylated genes, and the differential methylation was enriched in the 6th and 7th bins (Fig. 3c). Therefore, we can conclude that abnormal DNA methylation in the 6th and 7th bins of the enhancer region affects the expression of the DME gene.

### GO, KEGG analysis of D-Hyper, U-Hypo, and DME genes

**Fig. 4:**
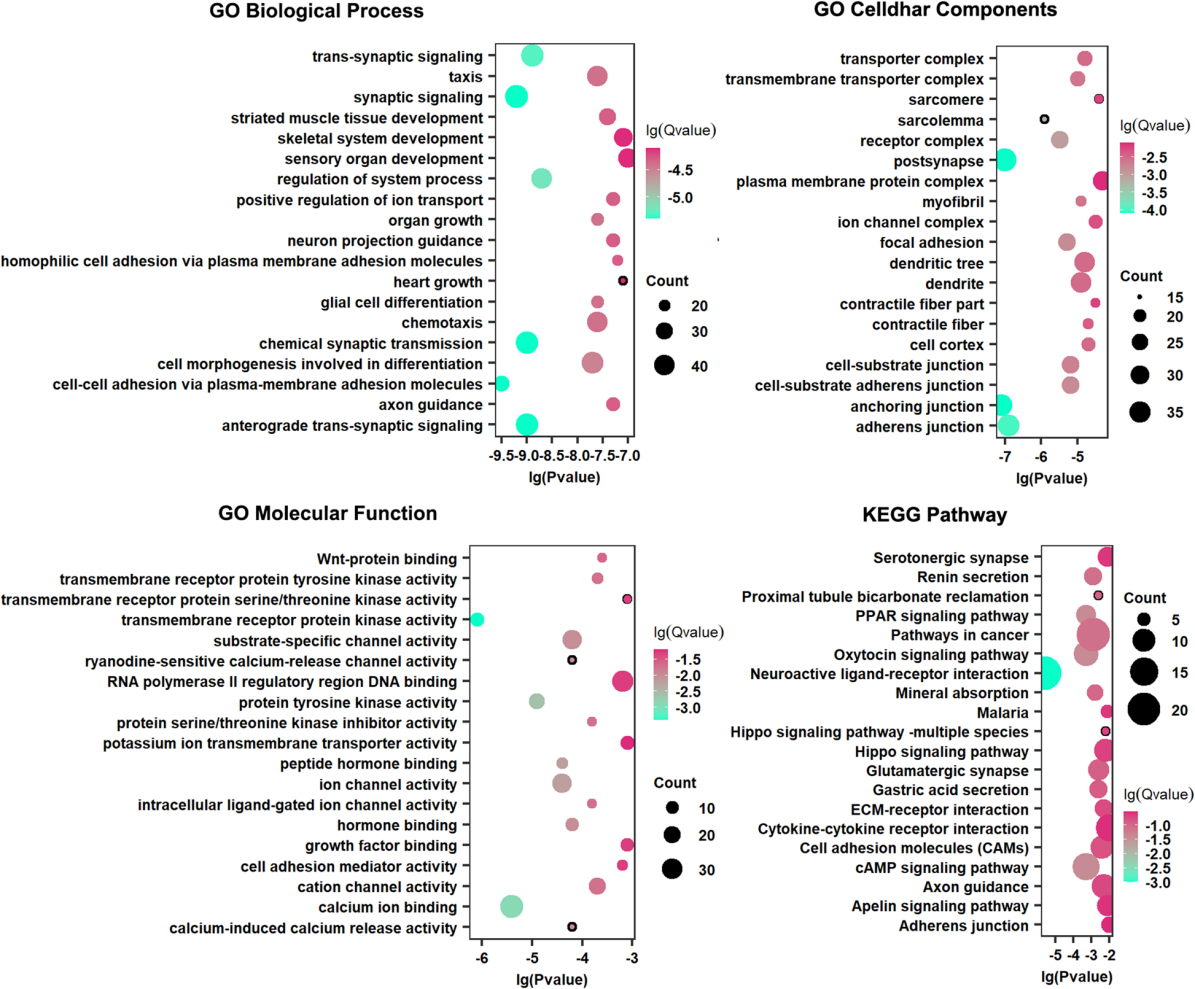
GO-function and KEGG pathway of D-Hyper genes. Bubble plot shows GO and KEGG pathway enrichment data for D-Hyper genes

To explore the potential biological functions of D-Hyper, U-Hypo, and DME genes, we performed GO function and KEGG pathway analysis. The GO analysis results showed that D-Hyper genes are related to the cell-cell adhesion via plasma-membrane adhesion molecules, regulation of system process, ion channel activity, RNA polymerase II regulatory region DNA binding, and growth factor binding, etc. The analysis of the KEGG pathway revealed that these genes were significantly enriched in Neuroactive ligand-receptor interaction, Oxytocin signaling pathway, and Pathways in cancer, etc. (Fig. 4). For U-Hypo genes, it was mainly related to the cytokine-mediated signaling pathway and the regulation of gene silencing. Transcriptional misregulation in cancer and the IL-17 signaling pathway are pathways associated with potential oncogenes (Supplementary Figure 1). The DME genes were mainly associated with the regulation of the ERBB signaling pathway, the negative regulation of cell differentiation, and the negative regulation of cell proliferation, etc. Through the KEGG pathway analysis revealed that the DME genes are enriched in pathways such as Focal adhesion, Viral carcinogenesis, and Melanoma (Supplementary Figure 2). These analyses suggested that the three kinds of the gene are relevant to cancer.

### The key genes related to breast cancer

**Fig. 5:**
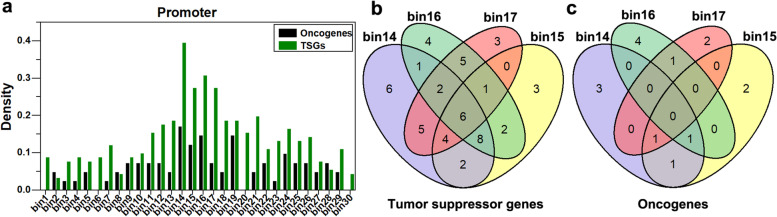
The distribution in promoter region for TSGs and oncogenes. **a** The distribution of density in promoter region for TSGs (tumor suppressor genes) and oncogenes. **b** and **c** Is the number of the tumor suppressor genes and oncogenes for abnormal DNA methylation in the promoter 14 ~ 17th bins

In order to further explore the DNA methylation distribution characteristics of oncogenes and TSGs, we intersect D-Hyper, U-Hypo, DME genes with cancer-related gene sets, respectively, and obtained 41 oncogenes and 91 TSGs. By analyzing the DNA methylation distribution of these genes, it can be seen that abnormal DNA methylation in the promoter region was still significantly enriched in 14 ~ 17th bins (− 200 bp, 200 bp) (Fig. 5a). Therefore, we further selected the genes of abnormal DNA methylation in the promoter 14 ~ 17th bins (− 200 bp, 200 bp) (Fig. 5b and c). Then by analyzing the GO and KEGG pathways of these genes, 20 key genes related to breast cancer were finally obtained, including 19 TSGs (*ACVR2A*, *CAV1*, *EGFR*, *FAT4*, *ID1*, *ID4*, *KIT*, *LEP*, *LEPR*, *MET*, *NRG1*, *PPARG*, *PRDM16*, *PREX2*, *PROX1*, *RYR3*, *SOX17*, *STAT5A*, *VIM*) and 1 oncogene (*PLK1*).

### The analysis of DNA methylation and gene expression for key genes in large samples

**Fig. 6:**
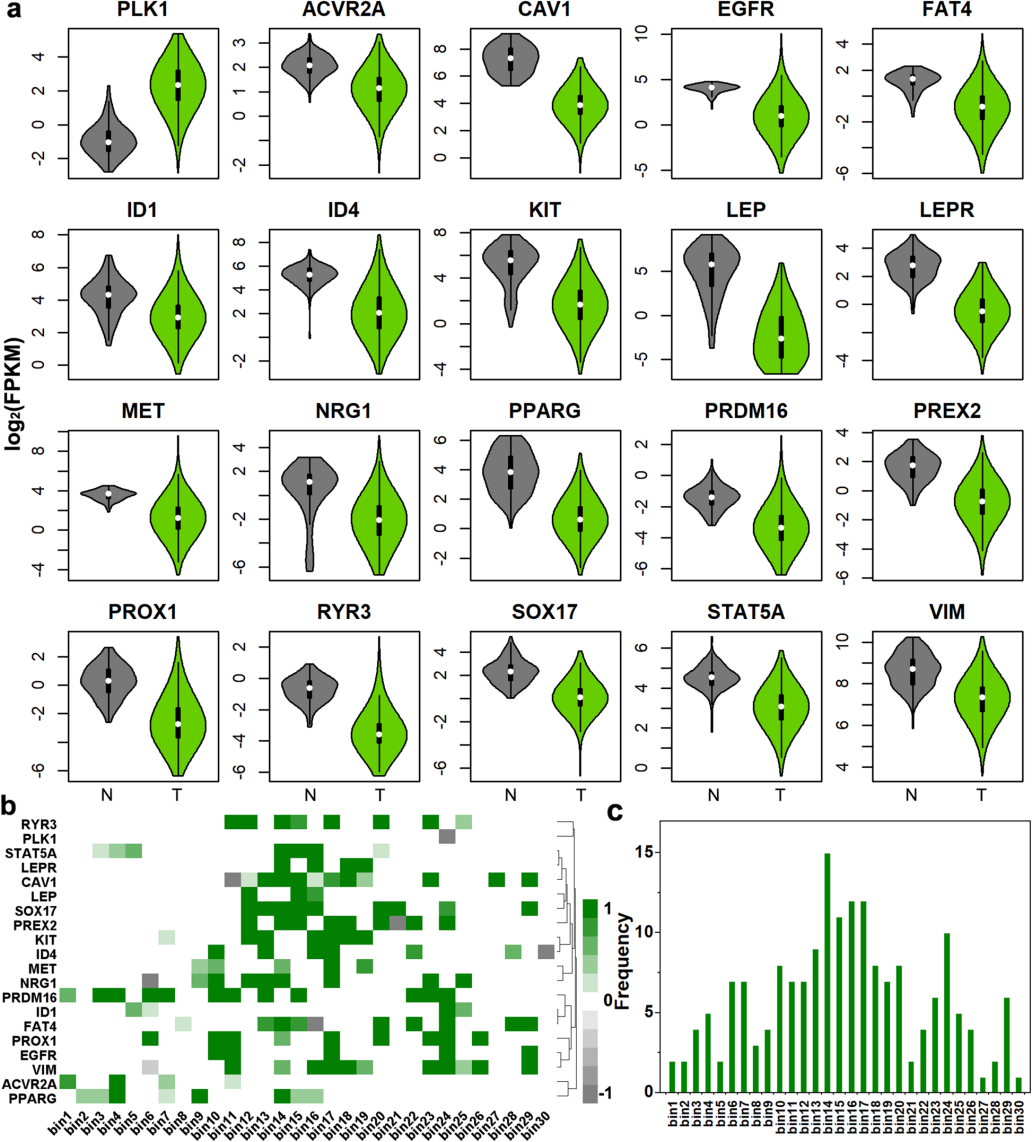
Gene expression levels and abnormal DNA methylation distribution in the promoter of 20 key genes. **a** The distributions of log_2_(FPKM) values of 20 key genes in cancer and paracancerous tissues. Dark gray is the distribution of log_2_(FPKM) values for paracancerous tissues, and light gray is the distribution of log_2_(FPKM) values for cancer tissues. **b** The distribution of the DNA methylation *RD* values for 30 bins in the promoter region. **c** The gene number distribution of 30 bins in the promoter region with an absolute value of *RD* greater than 0.2

To verify whether the 20 key genes had the same pattern in large samples, we analyzed DNA methylation data and gene expression data from breast cancer samples in TCGA. The genes expression levels (log_2_(FPKM)) of 20 are shown in Fig. 6a, it can be seen that the expression levels of the 19 genes were higher in the paracancerous tissues than in breast cancer tissues, except that the expression level of the *PLK1* was lower in the paracancerous tissues than in breast cancer tissues. It could be concluded that *PLK1* acts as an oncogene in breast cancer, while the remaining 19 genes acted to inhibit tumors. Figure 6b and c show the distribution of abnormal DNA methylation in the promoter regions of the 20 key genes in the large samples. The results indicated that only the 29th bin in the promoter region of the *PLK1* gene was abnormally hypomethylated, while other genes were abnormally hypermethylated (Fig. 6b). Figure 6c shows that abnormal DNA methylation in the promoter region was mainly enriched in 14 ~ 17th bins (− 200 bp, 200 bp). These are consistent with the conclusions we have drawn in the small sample.

### Survival analysis of key genes

**Fig. 7:**
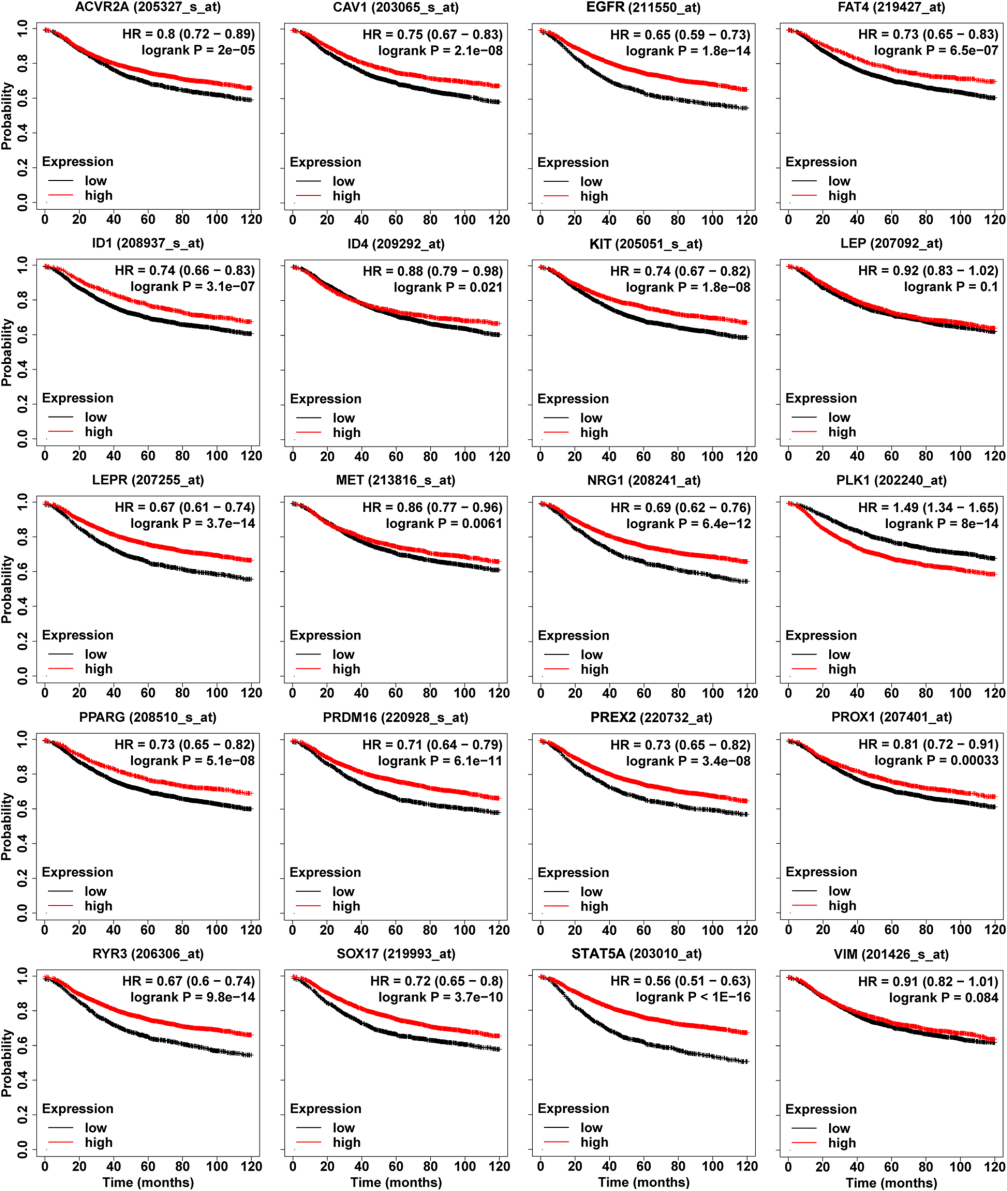
The effect of changes in expression levels of the 20 key genes on overall survival. The KM plotter of these key genes. The name of each figure is the gene symbol and Affymetrix ID. The abscissa is the survival time, the unit is the month, and the ordinate is the survival rate

To further verify the influence of the expression for the above 20 key genes on breast cancer, we used KM plotter to perform Kaplan-Meier survival analysis of these key genes to determine the prognostic value of these key genes in breast cancer. Figure 7 shows survival curves for all breast cancer patients (*n* = 4929). It can be seen that the high expression of *PLK1* was associated with poor overall survival of breast cancer patients. This result further indicated that *PLK1* was an oncogene. In contrast, the high expression of the 17 genes (*EGFR* (211550_at), *ACVR2A* (205327_s_at), *CAV1* (203065_s_at), *FAT4* (219427_at), *ID1* (208937_s_at), *ID4* (209292_at), *KIT* (205051_s_at), *LEPR* (207255_at), *MET* (213816_s_at), *NRG1* (208241_at), *PPARG* (208510_s_at), *PRDM16* (220928_s_at), *PREX2* (220732_at), *PROX1* (207401_at), *RYR3* (206306_at), *SOX17* (219993_at), and *STAT5A* (203010_at)) could significantly improve the prognosis of breast cancer patients. However, the high expression of *LEP* (207092_at) and *VIM* (201426_s_at) were weakly correlated with a good prognosis. It has been proved that 17 TSGs and 1 oncogene can be used as the markers of the prognosis for breast cancer patients.

## Discussion

Based on the analysis of the genome DNA methylation profiles for differentially expressed genes and DME genes, the results indicated that the most of hyper- and hypo- DNA methylation sites were located in the 14 ~ 17th bins (− 200 bp, 200 bp). Furthermore, we identified 1 oncogene and 17 TSGs as potential biomarkers for breast cancer diagnostic.

In addition, we further confirmed the selected TSGs and oncogenes are associated with breast cancer. We found that *EGFR*, *NRG1*, and *STAT5A* were enriched in the ErbB signaling pathway, *ID1* and *ID4* were enriched in the Hippo signaling pathway, *EGFR*, *KIT*, and *MET* were enriched in the PI3K-Akt signaling pathway and *SOX17* was enriched in the Wnt signaling pathway. These pathways related to environmental information processing and signal transduction had been shown to be closely related to the occurrence of breast cancer and play a decisive role [24, 26–29]. *EGFR*, *KIT*, *MET*, *PPARG*, and *STAT5A* were enriched in the Pathways in cancer, *CAV1*, *EGFR*, and *MET* were enriched in the Proteoglycans in cancer, and *MET* and *PPARG* were enriched in the Transcriptional misregulation in cancer. These pathways play a very important role in most human cancers [26, 30]. These findings further indicate that these genes can be used as biomarkers for breast cancer. In addition, *ACVR2A*, *ID1*, and *ID4* are enriched in the TGF-β signaling pathway, and the role of TGF-β in osteolytic bone metastasis was well known [31, 32]. The GO biological processes of *ACVR2A*, *KIT*, and *FAT4* are skeletal system development, *RYR3* and *EGFR* are in the Calcium signaling pathway enrichment, the GO molecular functions of *RYR3* and *FAT4* are calcium ion binding and the GO molecular function of *RYR3* is calcium-induced calcium release activity [33], and the most common metastasis of breast cancer is bone metastasis, so the enrichment of these GO biological processes, GO molecular functions and key pathways seems to explain why 85% of patients with advanced breast cancer have bone metastases [31, 34].

## Conclusions

In this study, we found that the abnormal DNA methylation of TSGs and oncogenes associated with the pathogenesis of breast cancer is mainly concentrated in the TSS ±200 bp region. we obtained 17 TSGs and 1 oncogene associated with breast cancer, and verified them by survival analysis. Our results may provide help for studying, the pathogenesis of breast cancer, potential therapeutic targets, and epigenetic modification as novel target drugs.

## Materials and methods

### Data sources

We downloaded the DNA methylation (Illumina Infinium Human Methylation 450 K) data and gene expression data (FPKM and COUNTS) of 1097 breast cancer samples from TCGA (https://tcga-data.nci.nih.gov/tcga/) (Table 1). There were 9 breast cancer samples that contain breast cancer tissue and matched paracancerous tissue data. The RefSeq genes of the human genome (hg38) were downloaded from the University of California Santa Cruz (UCSC) (http://genome.ucsc.edu/). The Ensemble Gene ID (GRCh38) annotation file and the location file of the human breast tissue enhancer were downloaded from Ensembl (http://www.ensembl.org/Homo_sapiens/).

**Table 1:** The details of 1097 breast cancer samples data types

Type	DNA methylation	Gene expression (FPKM and COUNTS)
Paracancerous sample	96	113
Cancer sample	789	1097

We obtained a cancer genes sets associated with breast cancer from the Catalogue Of Somatic Mutations In Cancer (COSMIC) (https://cancer.sanger.ac.uk/census) [35], the Candidate Cancer Gene Database (CCGD) (http://ccgd-starrlab.oit.umn.edu/download.php) [36], the Disease-gene associations mined from literature (DISEASES) (https://diseases.jensenlab.org) [37] and the National Cancer Institute (NCI) (https://wiki.nci.nih.gov/x/hC5yAQ).

### Selection of differentially expressed genes

To obtain the differentially expressed genes between the paracancerous tissue and the cancer tissue, first, we standardized the expression data used the following formulas.

1 $${NC}_{kj}=R\left({C}_{kj}/{s}_j\right)\kern0.5em \left(\begin{array}{cc}1\le k\le m,& 1\le j\le n\end{array}\right)$$

where

2 $${\displaystyle \begin{array}{l}{s}_j={e}^{\eta_j}\\ {}{\eta}_j=M\left(\begin{array}{cccc}{d}_{1j},& {d}_{2j},& \cdots, & {d}_{kj}\end{array}\right)\\ {}{d}_{kj}=\ln {C}_{kj}/\sqrt[n]{\prod \limits_{j=1}^n{C}_{kj}}\end{array}}$$

Here *k* is the *k-th* gene, *j* is the *j-th* sample, *NC*_*kj*_ denotes the normalized expression value of the *k-th* gene in the *j-th* sample, *R* denotes the rounding, *C*_*kj*_ is the gene expression counts of the *k-th* gene in the *j-th* sample, *s*_*j*_ is the standardization factor of the *j-th* sample, *m* (*m* = 60,483) is the total number of genes, and *n* (*n* = 1210) is the total number of samples, *M* denotes to take the median.

Then, using the DESeq function to calculate the differential expression of gene, got the log2(FoldChange), pval (*p* value for the statistical significance of this change) and padj (p value adjusted for multiple testing with the Benjamini-Hochberg procedure) [38]. Among them, Fold Change (*FC*) was calculated as follows:

3 $${FC}_k=\frac{\sum \limits_{j=1}^{n_t}{NC}_{kj,t}}{n_t}/\frac{\sum \limits_{j=1}^{n_p}{NC}_{kj,p}}{n_p}$$

Here, *k* and *j* are the same as Eq. (1), *t* is the cancer sample, *p* is the paracancerous sample, *NC*_*kj*, *t*_ (*NC*_*kj*, *p*_) is the normalized expression value of the *k-th* gene in the *j-th* cancer sample (paracancerous sample), *n*_*t*_ (*n*_*t*_ = 1097) is the number of the cancer sample, *n*_*p*_ (*n*_*p*_ = 113) is the number of the paracancerous sample. *FC*_*k*_ denotes the fold change of the *k-th* gene, when *FC*_*k*_ > 1.25, padj < 0.05 as up-regulated expression genes, and *FC*_*k*_ < 0.8, padj < 0.05 as down-regulated expression genes.

### Selection of differential DNA methylation genes

The DNA methylation levels of DNA methylation HM450K data were measured by the value of each probe. The degree of methylation (*β*) was defined as follows:

4 $$\begin{array}{cc}\beta_i=\frac{\max\left(y_{i,methy},0\right)}{\max\left(y_{i,methy},0\right)+\max\left(y_{i,umethy},0\right)+\alpha}&\left(1\leq i\leq m_w\right)\end{array}$$

Where *i* is the *i-th* CpG probe, *m*_*w*_ (*m*_*w*_ = 485,578) is the total number of the probes, max(*y*_*i*, *methy*_, 0) and max(*y*_*i*, *umethy*_, 0) are the signal intensities of the methylated and unmethylated for the *i-th* probe, respectively. The *α* is a constant (the default value is set to 100) to eliminate the effect on the *β* value when the max(*y*_*i*, *methy*_, 0) and max(*y*_*i*, *umethy*_, 0) are simultaneously low [39]. There are 485,578 probes in the file of the DNA methylation data, and 482,421 CpG probes were left after the non-CpG probes were removed. There are many “NA”s in the column due to the presence of single nucleotide polymorphisms (SNPs) [40]. Further, 96,079 probes containing “NA” were deleted. Finally, 386,342 probes remained.

We matched these probes to the promoter region (1500 bp upstream and downstream of TSS) of 18,861 genes, and calculated the DNA methylation level of genes according to the following formula.

5 $${\displaystyle \begin{array}{ll}{\beta}_{k,S}=\frac{\sum \limits_{i=1}^{m_r}{\beta}_{i,S}}{m_r}& \left(\begin{array}{cc}1\le i\le {m}_r,& S=t,p\end{array}\right)\\ {}{\beta}_{i,S}=\frac{\sum \limits_{j=1}^{n_S}{\beta}_{ij,S}}{n_S}& \left(1\le j\le {n}_S\right)\end{array}}$$

Here *i* is the same as Eq. (4), *k* and *j* are the same as Eq. (1), *S* is the sample type, *t* is the cancer sample, *p* is the paracancerous sample, *m*_*r*_ is the number of probes falling into the promoter region for the *k-th* gene, *β*_*i*, *S*_ is the average DNA methylation value of the *i-th* probe in the cancer sample (paracancerous sample), *β*_*k*, *S*_ is the DNA methylation level of the *k-th* gene in the cancer sample (paracancerous sample), *β*_*ij*, *S*_ denotes the DNA methylation level of the *i-th* probe in the *j-th* cancer sample (paracancerous sample), *n*_*S*_ is the total number of sample (*n*_*t*_ = 789, *n*_*p*_ = 96).

The genes of differential DNA methylation were defined by using relative difference (*RD*) [41]. The formula was as follows:

6 $${\displaystyle \begin{array}{cc}{RD}_k=\frac{\beta_{k,t}-{\beta}_{k,p}}{\beta_{k,p}+\Delta}& \left(1\le k\le {m}_g\right)\end{array}}$$

Here *k* is the *k-th* gene, *RD*_*k*_ is the relative difference of DNA methylation in the *k-th* gene, *β*_*k*, *t*_ and *β*_*k*, *p*_ denotes the average DNA methylation level of the *k-th* gene in the cancer sample and paracancerous sample, respectively. *Δ* = 10^−9^, *m*_*g*_ (*m*_*g*_ = 18,861) is the total number of the genes. When *RD*_*k*_ > 0.2, the DNA methylation level of this gene is higher in breast cancer tissues than that in paracancerous tissues, we defined it as a hypermethylated gene; when *RD*_*k*_ < − 0.2, the DNA methylation level of this gene is lower in breast cancer tissues than that in paracancerous tissues, we defined it as a hypomethylated gene.

### Calculation of DNA methylation levels in different regions

First, we used the file of tissue-specific gene regulatory location in the Ensembl database to extract the enhancer region. And according to the RefSeq annotation file, we divided the gene into the following regions: (1) promoter (TSS ± 1500 bp), (2) 5’UTR, (3) exon, (4) intron, (5) 3’UTR, (6) intergenic region. Second, the promoter region was divided into 30 bins, each bin was 100 bp, and the other genomic functional regions were divided into 10 bins, respectively. Third, the CpG site was matched to the bins, the methylation level of each bin was calculated by the following formula:

7 $${\displaystyle \begin{array}{cc}{\beta}_{k,S}^{\mu \xi}=\frac{\sum \limits_{i=1}^{m_r}{\beta}_{i,S}}{m_r}& \left(\begin{array}{cccc}1\le i\le {m}_w,& 1\le \mu \le {m}_b,& 1\le k\le {m}_g,& S=t,p\end{array}\right)\end{array}}$$

Here *k*, *i*, and *β*_*i*, *S*_ are the same as Eq. (5), *μ* is the *μ*-*th* bin, *ξ* is the *ξ*-*th* region, *m*_*w*_ (*m*_*w*_ = 386,342) is the total number of probes, *m*_*b*_ (*m*_*b*_ = 10) is the total number of bins, *m*_*g*_ (*m*_*g*_ = 18,861) is the total number of genes, *S* is the sample type, *t* is the cancer sample, *p* is the paracancerous sample, ${\beta}_{k,S}^{\mu \xi}$ is the DNA methylation level of the *μ-th* bin in the *ξ-th* region for the *k-th* gene. *m*_*r*_ is the number of probes falling into the *μ-th* bin in the *ξ-th* region for the *k-th* gene.

### Identifying the target gene of the enhancer and predicting genes regulated by differentially methylated enhancers (DME)

**Fig. 8:**
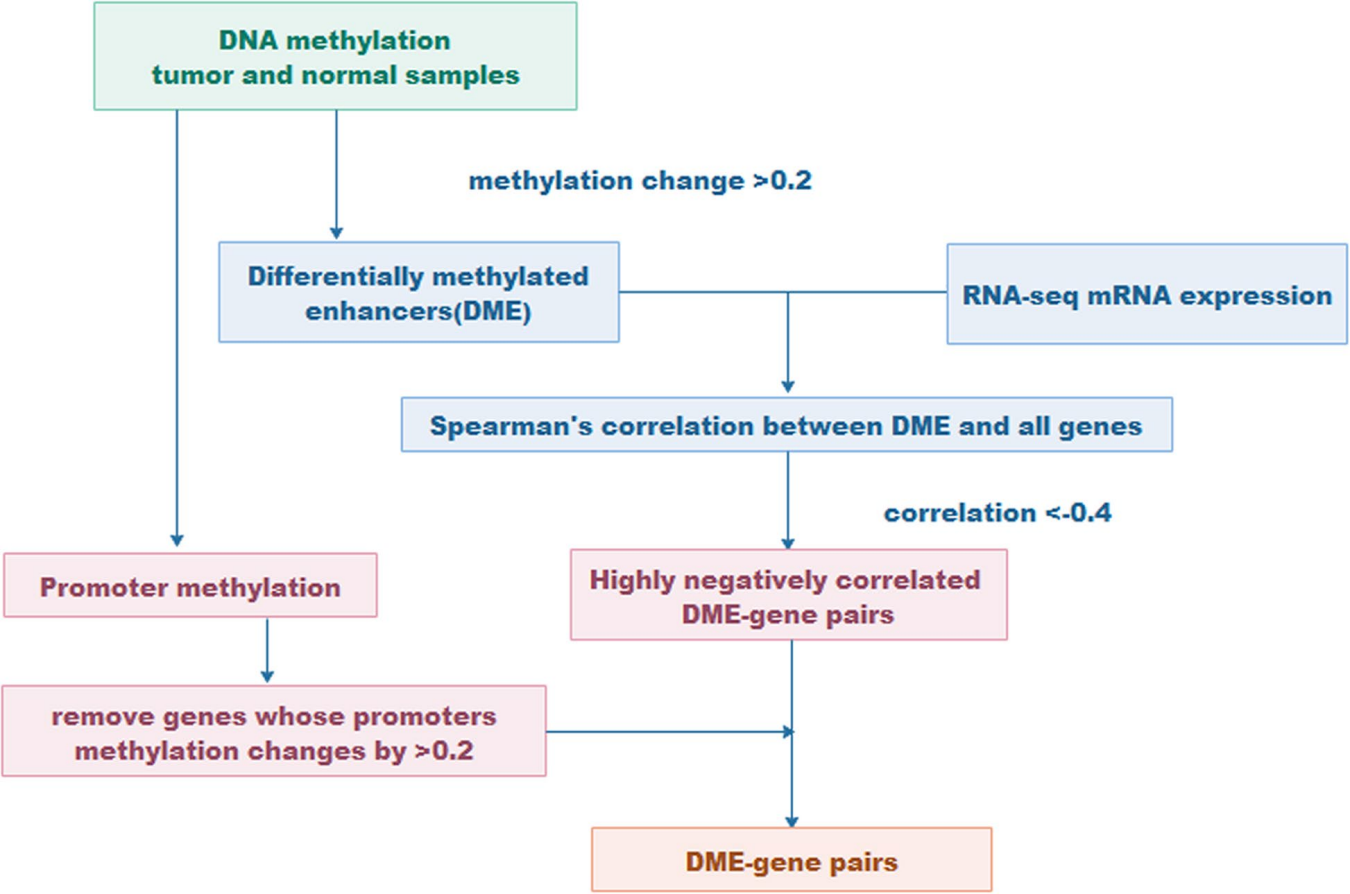
Flow chart of DME-gene pairs prediction

It is well known that the enhancer is a short (50 ~ 1500 bp) DNA region upstream or downstream 1Mbp of TSS in the gene [42, 43]. The enhancer can usually regulate the closest gene. By computing the distance from the TSS of each gene to the center of the enhancer region, and the gene closest to each enhancer was defined as the target gene of the enhancer. A comprehensive model was used for predicting genes regulated by DME (Fig. 8). First, we used Eq. (6) to calculate the differential methylation between the paracancerous tissue and the cancer tissue for the enhancer and promoter regions. Second, Spearman’s correlation (*r*_*k*_) between the relative difference (*RD*_*kj*_) of the DNA methylation for DME and the differential expression foldchange (*FC*_*kj*_) for its target gene was calculated in matched patients, and the highly negatively correlated (*r*_*k*_ < − 0.4) DME gene pairs were retained. Third, we removed the gene whose differential methylation value is greater than 0.2 in promoter, and obtained the differentially expressed genes only due to methylation changes in enhancer as the DME gene pairs [44]. Among them, the correlation coefficient was calculated using the following formula:

8 $${\displaystyle \begin{array}{cc}{r}_k=1-\frac{6\sum \limits_{j=1}^n{\left[R\left({FC}_{kj}\right)-R\left({RD}_{kj}\right)\right]}^2}{n\left({n}^2-1\right)}& \left(\begin{array}{cc}1\le j\le n,& 1\le k\le {m}_g\end{array}\right)\end{array}}$$

Where *k* and *j* are the same as Eq. (1), *n* (*n* = 9) is the total number of samples, *m*_*g*_ (*m*_*g*_ = 1902) is the total number of the target genes, *R*(*FC*_*kj*_) is the rank of differential expression foldchange for the *k-th* DME target gene. *R*(*RD*_*kj*_) is the rank of relative difference for methylation in the *k-th* DME.

### Gene ontology (GO) function, KEGG pathway, and survival analysis

GO and KEGG pathway enrichment analysis was performed using Metascape (http://metascape.org) [45]. Survival analysis was performed by using Kaplan-Meier Plotter (http://kmplot.com/). The Kaplan-Meier plotter has the information of 54,675 probes on survival using 5143 breast cancer patients with a mean follow-up of 69 months. Gene expression data and over survival (OS) information were downloaded from European Genome-phenome Archive (EGA), Gene Expression Omnibus (GEO), and TCGA [46].

## Supplementary Information

**Additional file 1: Supplement table.** Lists of eDMR-gene pair predictions.

**Additional file 2: Supplementary Figure 1.** GO-function and KEGG pathway of U-Hypo genes in breast cancer. **Supplementary Figure 2.** GO-function and KEGGpathway of DME genes in breast cancer.

## Data Availability

We have used the gene expression data, DNA methylation data and clinical data from TCGA database (https://tcga-data.nci.nih.gov/tcga/), known cancer genes from Catalogue Of Somatic Mutations In Cancer (COSMIC) (https://cancer.sanger.ac.uk/cosmic), the Candidate Cancer Gene Database (CCGD) (http://ccgd-starrlab.oit.umn.edu/download.php), the Disease-gene associations mined from literature (DISEASES) (https://diseases.jensenlab.org), and the National Cancer Institute (NCI) (https://wiki.nci.nih.gov/x/hC5yAQ), the genomic data from the UCSC database (http://genome.ucsc.edu/cgi-bin/hgTables) and Ensembl (http://www.ensembl.org/Homo_sapiens/), and the location file of enhancer from Ensembl (http://www.ensembl.org/Homo_sapiens/). These data are publicly available.

## Abbreviations

D-Hyper: Down-regulated and hypermethylated
eDMR: Differentially methylated enhancers
TSGs: Tumor suppressor genes
TSS: Transcription start site
U-Hypo: Up-regulated and hypomethylated
