# Supplementary material for: Discovering the key genes and important DNA methylation regions in breast cancer
Source: Hereditas. 2022 Jan 21;159:7. doi: 10.1186/s41065-022-00220-5 (PMC8781361; doi:10.1186/s41065-022-00220-5)
Supplement: Supplementary file 1 — Additional file 1: Supplement table. Lists of eDMR-gene pair predictions. [file 41065_2022_220_MOESM1_ESM.docx]

**Supplement table**

**Table: Lists of eDMR-gene pair predictions**

| eDMR | Gene symbols | chr | Distancer from TSS | Promoter differential methylation | Enhancer differential methylation | Differential gene expression | Correlation |
| --- | --- | --- | --- | --- | --- | --- | --- |
| ENSR00000162398 | KCNMB2 | chr3 | -734,198.500 | -0.110 | 0.752 | 0.354 | -0.405 |
| ENSR00000027048 | ANKRD30A | chr10 | -635,555.500 | -0.012 | 0.308 | 4.796 | -0.595 |
| ENSR00000026703 | PARD3 | chr10 | -405,458.500 | -0.037 | 0.479 | 0.606 | -0.524 |
| ENSR00000061609 | FOXO1 | chr13 | -363,462.000 | 0.031 | -0.289 | 0.489 | -0.405 |
| ENSR00000163584 | TPRG1 | chr3 | -317,572.500 | -0.081 | 0.312 | 6.993 | -0.690 |
| ENSR00000144758 | MN1 | chr22 | -312,076.000 | 0.000 | 0.984 | 2.488 | -0.429 |
| ENSR00000182558 | ENC1 | chr5 | -300,205.000 | 0.000 | -0.214 | 4.243 | -0.595 |
| ENSR00000000795 | VAMP3 | chr1 | -299,867.500 | 0.004 | 0.213 | 0.793 | -0.786 |
| ENSR00000070061 | ZFP36L1 | chr14 | -265,954.000 | 0.069 | 0.254 | 0.779 | -0.429 |
| ENSR00000007214 | FGGY | chr1 | -252,776.500 | 0.001 | 1.030 | 0.767 | -0.405 |
| ENSR00000186138 | ZNF608 | chr5 | -234,715.500 | 0.113 | 0.250 | 0.697 | -0.690 |
| ENSR00000033632 | HABP2 | chr10 | -226,476.500 | -0.019 | 0.833 | 1.752 | -0.643 |
| ENSR00000030372 | DLG5 | chr10 | -203,489.500 | 0.002 | -0.270 | 1.905 | -0.690 |
| ENSR00000179317 | RAI14 | chr5 | -187,205.500 | -0.050 | 0.332 | 1.887 | -0.524 |
| ENSR00000171720 | RPL34 | chr4 | -186,488.500 | -0.068 | 0.392 | 0.593 | -0.643 |
| ENSR00000043729 | CCDC82 | chr11 | -158,063.500 | 0.000 | 1.133 | 0.491 | -0.429 |
| ENSR00000205605 | TIAM2 | chr6 | -157,687.000 | -0.049 | 0.270 | 0.722 | -0.405 |
| ENSR00000132161 | ARL4C | chr2 | -157,639.500 | 0.074 | 2.191 | 0.643 | -0.857 |
| ENSR00000169525 | SOWAHB | chr4 | -155,750.500 | 0.002 | 0.267 | 2.522 | -0.429 |
| ENSR00000309980 | CHIC2 | chr4 | -146,590.000 | 0.000 | 0.456 | 0.785 | -0.476 |
| ENSR00000016547 | XPR1 | chr1 | -115,908.500 | -0.035 | 0.465 | 1.713 | -0.476 |
| ENSR00000089702 | SPG7 | chr16 | -113,186.000 | -0.031 | 0.618 | 0.782 | -0.429 |
| ENSR00000113681 | TTC32 | chr2 | -110,130.000 | 0.000 | -0.297 | 0.769 | -0.405 |
| ENSR00000330819 | SFRP1 | chr8 | -109,656.000 | 0.000 | -0.420 | 0.109 | -0.548 |
| ENSR00000223908 | SFRP1 | chr8 | -103,556.000 | 0.000 | 0.261 | 0.109 | -0.452 |
| ENSR00000046346 | GRAMD1B | chr11 | -91,134.500 | 0.130 | -0.277 | 0.799 | -0.452 |
| ENSR00000034272 | RGS10 | chr10 | -86,225.500 | 0.039 | 0.404 | 2.325 | -0.429 |
| ENSR00000036997 | MICALCL | chr11 | -86,198.500 | 0.198 | 0.649 | 8.659 | -0.524 |
| ENSR00000169369 | BTC | chr4 | -84,958.500 | 0.000 | 2.043 | 0.646 | -0.881 |
| ENSR00000123435 | TMEM163 | chr2 | -82,757.500 | 0.000 | 0.237 | 0.581 | -0.595 |
| ENSR00000176937 | CASP3 | chr4 | -79,994.500 | 0.001 | -0.274 | 1.532 | -0.476 |
| ENSR00000302802 | SLC6A6 | chr3 | -78,874.500 | 0.117 | 0.347 | 1.365 | -0.952 |
| ENSR00000033963 | ENO4 | chr10 | -78,110.000 | 0.177 | 0.637 | 1.339 | -0.690 |
| ENSR00000073474 | EXOC3L4 | chr14 | -68,442.500 | 0.015 | 0.798 | 2.460 | -0.500 |
| ENSR00000170521 | SPP1 | chr4 | -64,248.500 | -0.074 | -0.320 | 7.899 | -0.429 |
| ENSR00000046587 | RPUSD4 | chr11 | -62,193.000 | 0.000 | 0.635 | 0.759 | -0.571 |
| ENSR00000164069 | CPN2 | chr3 | -62,063.500 | -0.016 | 0.623 | 2,322,194.437 | -0.905 |
| ENSR00000201320 | RFPL4B | chr6 | -57,929.000 | -0.030 | 0.667 | 931,565.562 | -0.452 |
| ENSR00000089722 | SPG7 | chr16 | -49,386.500 | -0.031 | 0.237 | 0.782 | -0.405 |
| ENSR00000021155 | PSEN2 | chr1 | -49,070.500 | 0.062 | 0.405 | 1.723 | -0.595 |
| ENSR00000159070 | FOXL2 | chr3 | -45,822.000 | -0.061 | 0.859 | 428,866.284 | -0.548 |
| ENSR00000228253 | AZIN1 | chr8 | -43,300.000 | 0.041 | -0.268 | 1.403 | -0.476 |
| ENSR00000208618 | ETV1 | chr7 | -41,129.000 | 0.000 | -0.429 | 0.489 | -0.667 |
| ENSR00000240907 | DAB2IP | chr9 | -37,400.500 | -0.020 | 1.077 | 0.427 | -0.714 |
| ENSR00000036163 | RRM1 | chr11 | -36,583.500 | 0.048 | 1.876 | 1.278 | -0.690 |
| ENSR00000072509 | SERPINA1 | chr14 | -31,045.000 | 0.000 | -0.225 | 18.991 | -0.476 |
| ENSR00000080858 | ABHD2 | chr15 | -30,448.500 | 0.122 | 0.361 | 2.648 | -0.833 |
| ENSR00000186572 | FBN2 | chr5 | -29,707.500 | 0.016 | 0.591 | 6.922 | -0.524 |
| ENSR00000016825 | NMNAT2 | chr1 | -29,536.000 | 0.000 | 0.369 | 0.257 | -0.881 |
| ENSR00000119086 | ACTG2 | chr2 | -22,565.000 | 0.142 | 0.334 | 0.145 | -0.452 |
| ENSR00000177376 | LRRC14B | chr5 | -22,509.000 | -0.102 | 0.206 | 2.516 | -0.405 |
| ENSR00000032194 | ANKRD2 | chr10 | -22,339.500 | -0.075 | 0.332 | 3.213 | -0.690 |
| ENSR00000039609 | C11orf49 | chr11 | -22,087.500 | -0.034 | 0.468 | 1.392 | -0.500 |
| ENSR00000210909 | KIAA0895 | chr7 | -20,848.500 | -0.017 | 0.496 | 1.379 | -0.857 |
| ENSR00000128794 | TRAK2 | chr2 | -19,705.500 | 0.000 | 0.508 | 1.424 | -0.714 |
| ENSR00000086513 | CNGB1 | chr16 | -18,639.000 | 0.139 | 0.713 | 63,711.106 | -0.571 |
| ENSR00000149483 | SGO1 | chr3 | -18,491.500 | 0.000 | 0.505 | 22.480 | -0.571 |
| ENSR00000023150 | SCCPDH | chr1 | -18,474.500 | 0.081 | -0.369 | 2.727 | -0.452 |
| ENSR00000094812 | GJC1 | chr17 | -16,246.500 | 0.000 | 0.371 | 1.849 | -0.452 |
| ENSR00000010954 | SLC25A24 | chr1 | -16,120.500 | 0.000 | -0.345 | 1.344 | -0.452 |
| ENSR00000088733 | SLC38A8 | chr16 | -15,565.000 | 0.000 | -0.375 | 13,711,542.560 | -0.619 |
| ENSR00000019877 | PTPN14 | chr1 | -15,395.000 | 0.008 | 0.220 | 0.333 | -0.429 |
| ENSR00000068834 | TBPL2 | chr14 | -15,109.500 | 0.000 | 0.982 | 2,446,705.277 | -0.643 |
| ENSR00000058677 | OGFOD2 | chr12 | -12,801.500 | 0.016 | -0.290 | 1.413 | -0.524 |
| ENSR00000081000 | IDH2 | chr15 | -12,578.500 | -0.005 | 0.746 | 2.188 | -0.714 |
| ENSR00000003763 | IFI6 | chr1 | -10,059.000 | 0.000 | 1.049 | 11.154 | -0.452 |
| ENSR00000019018 | MAPKAPK2 | chr1 | -10,018.500 | 0.059 | 0.330 | 2.430 | -0.405 |
| ENSR00000243124 | FUT7 | chr9 | -7,872.500 | 0.068 | 0.259 | 6,536,262.719 | -0.429 |
| ENSR00000019388 | LAMB3 | chr1 | -7,172.000 | -0.028 | -0.345 | 0.268 | -0.524 |
| ENSR00000156757 | DRD3 | chr3 | -6,151.000 | 0.000 | -0.272 | 4,412,071.295 | -0.409 |
| ENSR00000035749 | MUC5AC | chr11 | -5,451.500 | -0.062 | -0.218 | 6,290,150.766 | -0.595 |
| ENSR00000087571 | COG4 | chr16 | -3,967.000 | 0.032 | -0.237 | 0.759 | -0.643 |
| ENSR00000190918 | STC2 | chr5 | -3,721.500 | -0.113 | 0.921 | 2.447 | -0.405 |
| ENSR00000132092 | HJURP | chr2 | -3,699.500 | 0.003 | 0.632 | 20.998 | -0.690 |
| ENSR00000066437 | RNASE7 | chr14 | -2,824.500 | 0.147 | 1.564 | 0.123 | -0.476 |
| ENSR00000016422 | TOR3A | chr1 | -2,675.500 | -0.073 | 0.598 | 2.035 | -0.571 |
| ENSR00000029372 | LRRC20 | chr10 | -2,268.500 | -0.171 | 0.431 | 2.035 | -0.810 |
| ENSR00000105543 | PTBP1 | chr19 | -1,990.500 | 0.037 | 0.255 | 1.400 | -0.429 |
| ENSR00000151389 | KLHL40 | chr3 | -1,717.500 | -0.004 | 0.699 | 923,641.176 | -0.738 |
| ENSR00000079696 | CIB2 | chr15 | -704.500 | -0.146 | -0.253 | 0.484 | -0.524 |
| ENSR00000133701 | C20orf141 | chr20 | -85.500 | -0.029 | -0.372 | 5,323,305.750 | -0.791 |
| ENSR00000093395 | CCL8 | chr17 | 54.500 | -0.200 | 0.321 | 1.922 | -0.452 |
| ENSR00000041665 | FOLR1 | chr11 | 143.500 | 0.087 | 0.844 | 0.162 | -0.476 |
| ENSR00000062264 | KCTD4 | chr13 | 1,948.000 | 0.149 | 0.599 | 0.035 | -0.405 |
| ENSR00000090182 | SMG6 | chr17 | 2,963.000 | -0.091 | 0.293 | 0.693 | -0.429 |
| ENSR00000137668 | PIGT | chr20 | 3,035.000 | -0.158 | -0.265 | 1.907 | -0.452 |
| ENSR00000119091 | ACTG2 | chr2 | 4,035.000 | 0.142 | 0.844 | 0.145 | -0.571 |
| ENSR00000178032 | CMBL | chr5 | 6,606.500 | 0.000 | 0.282 | 1.593 | -0.500 |
| ENSR00000193741 | JARID2 | chr6 | 7,347.000 | 0.099 | 0.257 | 1.379 | -0.452 |
| ENSR00000072440 | ASB2 | chr14 | 7,648.500 | -0.076 | 0.277 | 0.748 | -0.571 |
| ENSR00000143872 | RTN4R | chr22 | 8,686.000 | 0.009 | 0.663 | 9.762 | -0.810 |
| ENSR00000002369 | IFFO2 | chr1 | 9,121.500 | -0.047 | 0.814 | 0.264 | -0.690 |
| ENSR00000199895 | RRAGD | chr6 | 11,085.500 | 0.000 | -0.582 | 0.524 | -0.690 |
| ENSR00000130099 | IGFBP2 | chr2 | 11,197.500 | -0.065 | 1.270 | 2.726 | -0.619 |
| ENSR00000212642 | EGFR | chr7 | 11,284.500 | 0.056 | 0.359 | 0.204 | -0.476 |
| ENSR00000033753 | AFAP1L2 | chr10 | 11,877.500 | -0.020 | -0.206 | 0.672 | -0.619 |
| ENSR00000281694 | CDH13 | chr16 | 12,807.000 | 0.152 | -0.348 | 0.473 | -0.500 |
| ENSR00000186794 | CSF2 | chr5 | 12,809.500 | -0.135 | 0.923 | 522,301.949 | -0.898 |
| ENSR00000173878 | TBC1D9 | chr4 | 15,119.000 | -0.028 | 0.239 | 2.235 | -0.762 |
| ENSR00000096390 | CLTC | chr17 | 15,312.500 | 0.025 | 0.243 | 1.924 | -0.762 |
| ENSR00000004475 | TRIM62 | chr1 | 15,999.500 | 0.038 | 0.856 | 1.910 | -0.524 |
| ENSR00000041837 | P2RY2 | chr11 | 18,944.500 | -0.004 | -0.212 | 2.051 | -0.762 |
| ENSR00000084812 | KIAA0556 | chr16 | 22,354.500 | -0.138 | 0.775 | 1.803 | -0.905 |
| ENSR00000273980 | GPR33 | chr14 | 22,657.500 | 0.000 | 0.347 | 24,555,654.590 | -0.464 |
| ENSR00000029539 | SLC29A3 | chr10 | 24,348.500 | 0.090 | -0.245 | 2.831 | -0.643 |
| ENSR00000159968 | TM4SF1 | chr3 | 26,583.500 | 0.017 | -0.224 | 0.548 | -0.619 |
| ENSR00000111270 | FPR3 | chr19 | 28,955.500 | -0.158 | -0.237 | 4.293 | -0.429 |
| ENSR00000209433 | TOMM7 | chr7 | 29,868.500 | 0.009 | 0.318 | 0.676 | -0.429 |
| ENSR00000207751 | SNX8 | chr7 | 30,131.000 | -0.037 | 0.228 | 1.338 | -0.810 |
| ENSR00000052207 | ATF7 | chr12 | 30,745.500 | 0.021 | 0.695 | 0.730 | -0.452 |
| ENSR00000248598 | TENM1 | chrX | 30,995.500 | 0.000 | 0.354 | 0.373 | -0.476 |
| ENSR00000228912 | TRPS1 | chr8 | 31,605.500 | 0.000 | 0.277 | 2.535 | -0.476 |
| ENSR00000159969 | TM4SF1 | chr3 | 32,883.500 | 0.017 | -0.364 | 0.548 | -0.524 |
| ENSR00000188054 | FGF1 | chr5 | 33,323.500 | -0.027 | 0.842 | 0.255 | -0.452 |
| ENSR00000195643 | TNXB | chr6 | 35,346.500 | -0.062 | 0.232 | 0.158 | -0.405 |
| ENSR00000068374 | NID2 | chr14 | 39,099.500 | 0.011 | -0.334 | 4.080 | -0.405 |
| ENSR00000036503 | ST5 | chr11 | 39,649.500 | 0.000 | 0.518 | 0.672 | -0.595 |
| ENSR00000085575 | ABCC12 | chr16 | 40,428.500 | -0.010 | 0.269 | 311,818.397 | -0.667 |
| ENSR00000215101 | CDK6 | chr7 | 40,480.500 | 0.000 | -0.327 | 0.587 | -0.690 |
| ENSR00000186180 | ZNF608 | chr5 | 41,484.500 | 0.113 | 0.361 | 0.697 | -0.548 |
| ENSR00000037027 | MICALCL | chr11 | 44,502.000 | 0.198 | 0.716 | 8.659 | -0.524 |
| ENSR00000162013 | NCEH1 | chr3 | 46,156.500 | 0.004 | 0.504 | 1.661 | -0.762 |
| ENSR00000003160 | IFNLR1 | chr1 | 48,044.500 | 0.035 | 0.366 | 0.618 | -0.452 |
| ENSR00000157467 | MYLK | chr3 | 48,705.500 | 0.000 | 0.579 | 0.216 | -0.524 |
| ENSR00000210401 | INMT | chr7 | 48,767.000 | 0.080 | 0.330 | 0.529 | -0.857 |
| ENSR00000083334 | RMI2 | chr16 | 51,063.500 | -0.125 | 0.630 | 4.926 | -0.690 |
| ENSR00000088938 | FAM92B | chr16 | 53,649.500 | -0.135 | 1.020 | 7.945 | -0.690 |
| ENSR00000213530 | CLDN4 | chr7 | 55,738.000 | -0.152 | 0.801 | 1.712 | -0.500 |
| ENSR00000193095 | NEDD9 | chr6 | 58,504.000 | 0.000 | 0.522 | 0.563 | -0.548 |
| ENSR00000132976 | HDAC4 | chr2 | 65,933.500 | 0.001 | 1.373 | 0.671 | -0.595 |
| ENSR00000055842 | FGD6 | chr12 | 67,352.500 | 0.125 | 0.735 | 2.016 | -0.500 |
| ENSR00000021510 | URB2 | chr1 | 67,985.500 | -0.016 | 0.394 | 1.345 | -0.452 |
| ENSR00000077873 | DAPK2 | chr15 | 72,166.000 | 0.000 | 0.226 | 0.597 | -0.476 |
| ENSR00000006622 | SSBP3 | chr1 | 73,070.500 | 0.000 | 1.732 | 0.774 | -0.595 |
| ENSR00000172813 | SPRY1 | chr4 | 82,107.000 | 0.045 | -0.279 | 0.732 | -0.619 |
| ENSR00000157473 | MYLK | chr3 | 82,605.500 | 0.000 | 0.551 | 0.216 | -0.476 |
| ENSR00000122167 | ACTR3 | chr2 | 87,403.500 | 0.017 | 0.222 | 1.428 | -0.595 |
| ENSR00000245898 | BCOR | chrX | 90,055.000 | -0.080 | 1.072 | 1.777 | -0.643 |
| ENSR00000031935 | SORBS1 | chr10 | 97,428.500 | 0.000 | 0.364 | 0.690 | -0.524 |
| ENSR00000167793 | APBB2 | chr4 | 99,374.500 | -0.025 | -0.256 | 1.505 | -0.571 |
| ENSR00000089688 | ANKRD11 | chr16 | 102,480.500 | 0.042 | 0.467 | 0.665 | -0.571 |
| ENSR00000058875 | NCOR2 | chr12 | 103,691.000 | -0.003 | 0.217 | 1.252 | -0.524 |
| ENSR00000180928 | C5orf67 | chr5 | 104,807.500 | 0.000 | 0.948 | 0.514 | -0.810 |
| ENSR00000021567 | GALNT2 | chr1 | 104,906.000 | 0.089 | -0.252 | 1.338 | -0.690 |
| ENSR00000114625 | PLB1 | chr2 | 111,530.500 | -0.065 | 0.355 | 0.588 | -0.571 |
| ENSR00000007298 | FGGY | chr1 | 117,423.000 | 0.001 | -0.444 | 0.767 | -0.571 |
| ENSR00000031517 | ANKRD1 | chr10 | 122,701.500 | 0.000 | 1.961 | 7,048,424.843 | -0.476 |
| ENSR00000237407 | NXNL2 | chr9 | 123,300.500 | -0.141 | 0.301 | 4.028 | -0.667 |
| ENSR00000149811 | THRB | chr3 | 125,247.500 | 0.000 | 0.235 | 0.458 | -0.571 |
| ENSR00000167111 | STIM2 | chr4 | 132,410.500 | -0.063 | 1.353 | 0.688 | -0.476 |
| ENSR00000123251 | NCKAP5 | chr2 | 132,602.000 | -0.086 | 0.327 | 1.478 | -0.452 |
| ENSR00000153737 | ADAMTS9 | chr3 | 135,346.500 | 0.000 | -0.272 | 0.750 | -0.429 |
| ENSR00000028742 | ARID5B | chr10 | 139,947.500 | -0.119 | -0.211 | 0.418 | -0.571 |
| ENSR00000014817 | NOS1AP | chr1 | 143,611.000 | -0.104 | 0.348 | 1.639 | -0.548 |
| ENSR00000182476 | ARHGEF28 | chr5 | 200,243.500 | 0.033 | 0.333 | 0.436 | -0.643 |
| ENSR00000132395 | AGAP1 | chr2 | 208,913.000 | 0.026 | 0.328 | 0.795 | -0.881 |
| ENSR00000096125 | MSI2 | chr17 | 217,131.000 | 0.102 | 0.641 | 1.897 | -0.429 |
| ENSR00000203924 | CITED2 | chr6 | 235,646.500 | 0.059 | 0.353 | 0.733 | -0.524 |
| ENSR00000064652 | ABCC4 | chr13 | 260,772.500 | -0.105 | 0.248 | 1.727 | -0.452 |
| ENSR00000123003 | HS6ST1 | chr2 | 297,922.000 | -0.005 | 0.711 | 1.808 | -0.476 |
| ENSR00000308955 | BOD1L1 | chr4 | 351,759.000 | 0.000 | -0.281 | 0.797 | -0.405 |
| ENSR00000214475 | CACNA2D1 | chr7 | 373,457.500 | 0.000 | 0.231 | 1.337 | -0.452 |
