# Supplementary material for: Discovering the key genes and important DNA methylation regions in breast cancer
Source: Hereditas. 2022 Jan 21;159:7. doi: 10.1186/s41065-022-00220-5 (PMC8781361; doi:10.1186/s41065-022-00220-5)
Supplement: Supplementary file 2 — Additional file 2: Supplementary Figure 1. GO-function and KEGG pathway of U-Hypo genes in breast cancer. Supplementary Figure 2. GO-function and KEGGpathway of DME genes in breast cancer. [file 41065_2022_220_MOESM2_ESM.docx]

**
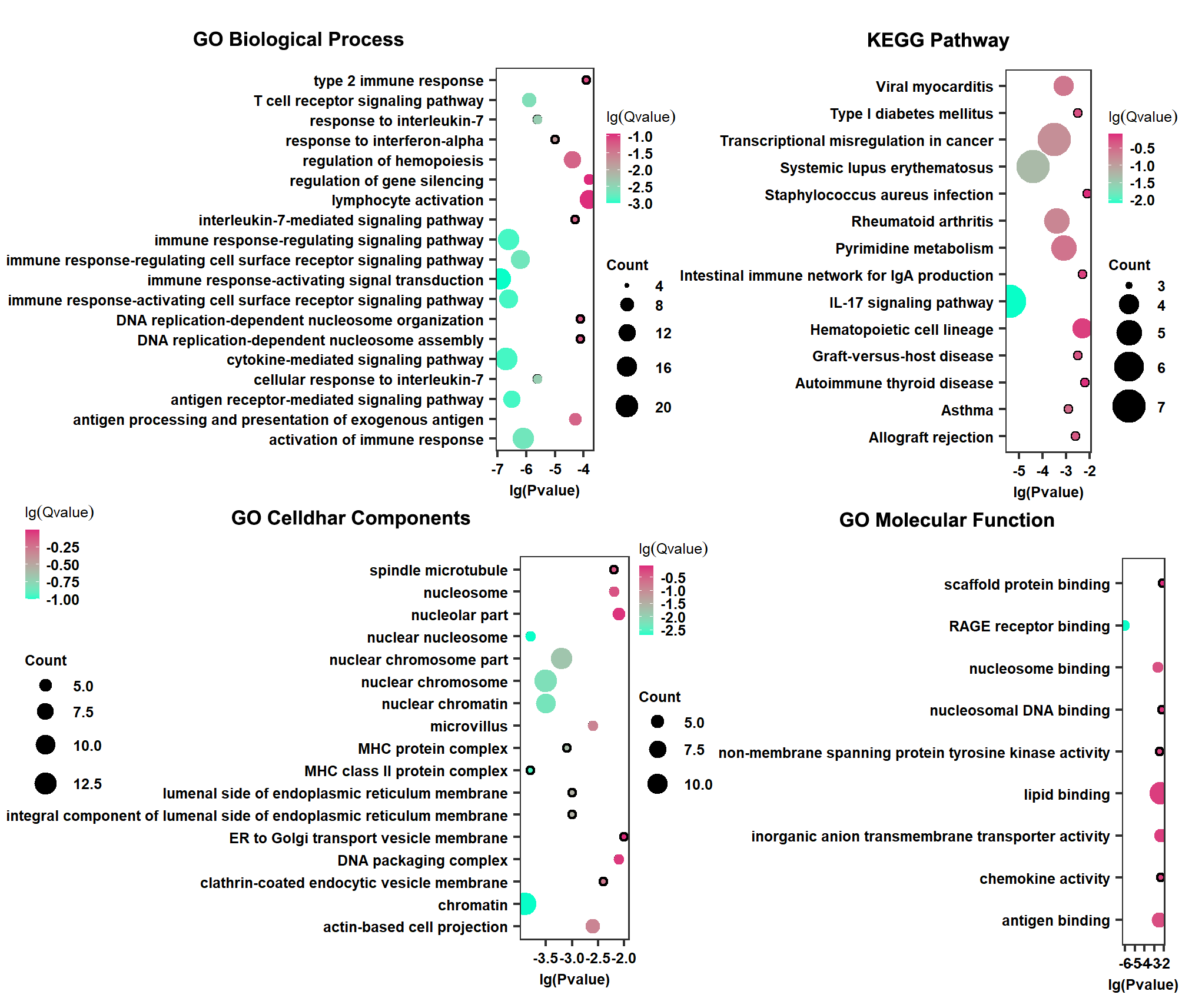
Supplementary Figure 1.** GO-function and KEGG pathway of U-Hypo genes in breast cancer.

**
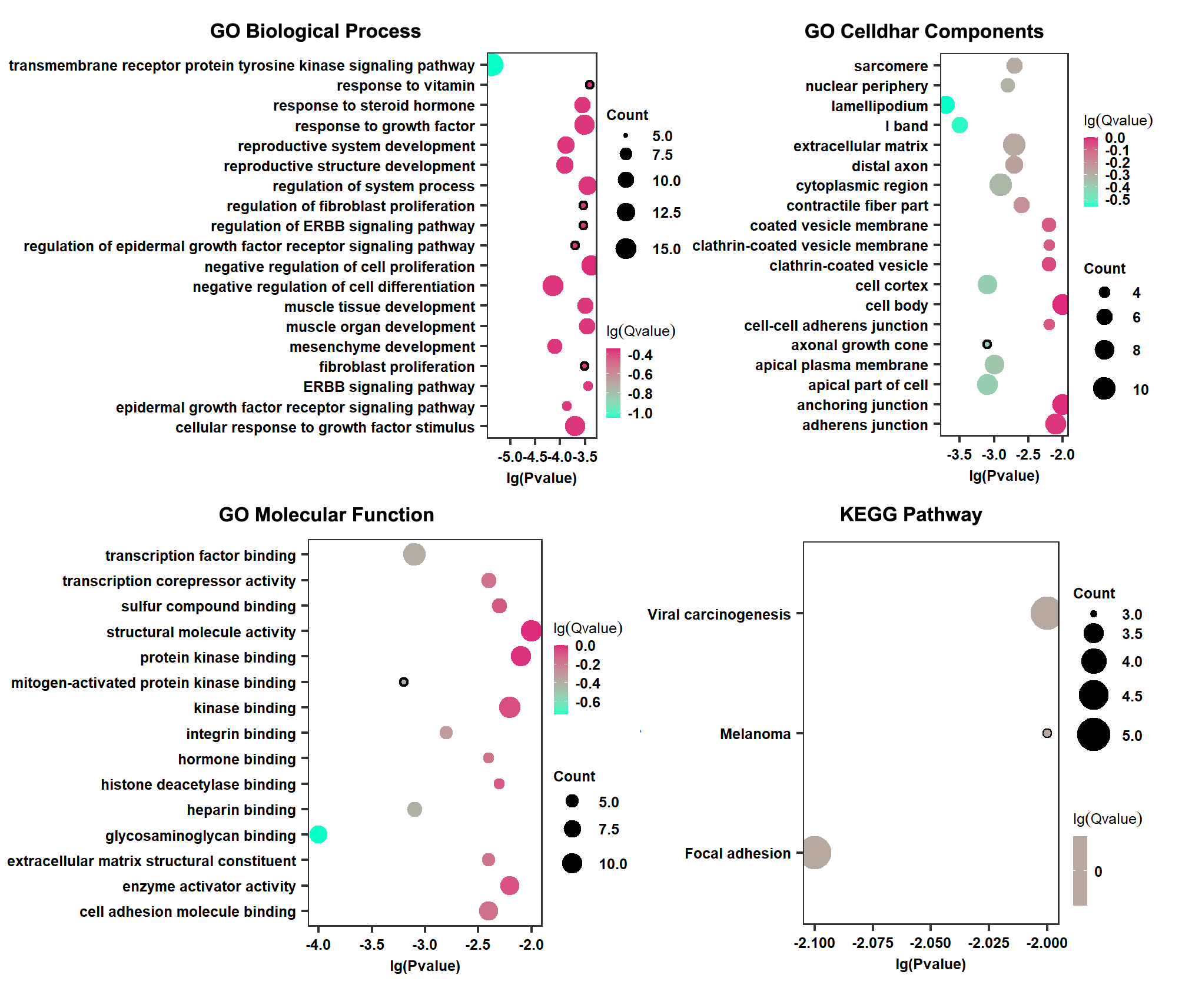
Supplementary Figure 2.** GO-function and KEGG pathway of DME genes in breast cancer.
